# Promoter methylation of PCDH10 by HOTAIR regulates the progression of gastrointestinal stromal tumors

**DOI:** 10.18632/oncotarget.12171

**Published:** 2016-09-21

**Authors:** Na Keum Lee, Jung Hwa Lee, Won Kyu Kim, Seongju Yun, Young Hoon Youn, Chan Hyuk Park, Yun Young Choi, Hogeun Kim, Sang Kil Lee

**Affiliations:** ^1^ Yonsei Institute of Gastroenterology, Department of Internal Medicine, Yonsei University College of Medicine, South Korea; ^2^ Department of pathology, Department of Internal Medicine, Yonsei University College of Medicine, South Korea

**Keywords:** PCDH10, HOTAIR, gastrointestinal stromal tumor

## Abstract

HOTAIR, a long non-coding RNA (lncRNA), plays a crucial role in tumor initiation and metastasis by interacting with the PRC2 complex and the modulation of its target genes. The role of HOTAIR in gastrointestinal stromal tumors (GISTs) is remains unclear. Herein we investigate the mechanism of HOTAIR in the genesis and promotion of GISTs. The expression of HOTAIR was found to be higher in surgically resected high-risk GISTs than that in low- and intermediate-risk GISTs. Using GIST-T1 and GIST882 cells, we demonstrated that HOTAIR repressed apoptosis, was associated with cell cycle progression, and controlled the invasion and migration of GIST cells. Using a gene expression microarray and lists of HOTAIR-associated candidate genes, we suggested that protocadherin 10 (PCDH10) is a key molecule. PCDH10 expression was significantly decreased in GIST-T1 and GIST882 cells, possibly as a consequence of hypermethylation. We observed that HOTAIR induced PCDH10 methylation in a SUZ12-dependent manner. In this study, we found that the malignant character of GISTs was initiated and amplified by PCDH10 in a process regulated by HOTAIR. In summary, our findings imply that PCDH10 and HOTAIR may be useful markers of disease progression and therapeutic targets.

## INTRODUCTION

Gastrointestinal stromal tumors (GISTs) are a type of mesenchymal tumor, and constitute the most common form of subepithelial tumor in the stomach [1, 2]. GIST cells present sporadic mutations in the c-KIT and PDGFRA genes, which encode the KIT and platelet-derived growth factor receptor alpha proteins, respectively. However, GISTs present an extremely heterogeneous clinical prognosis. Incidentally, most GISTs are detected via screening endoscopy. Though they usually remain clinically silent, some nevertheless progress to become malignant. Currently, the criteria for risk estimation depend largely on clinicopathologic factors, such as size and mitosis index, however these cannot accurately predict the risk of malignancy.

Long noncoding RNAs (lncRNAs) are non-coding transcripts longer than 200 nt, the amplification of which regulates gene expression, including genomic imprinting, cancer initiation and progression, and RNA turnover [3]. Homeobox (HOX) transcript antisense intergenic RNA (HOTAIR), a lncRNA, has been reported to regulate gene expression by modifying chromatin structure via the recruitment of polycomb repressive complex 2 (PRC2). This is composed of subunits EZH2, SUZ12, and EED, which are all involved in histone modification via interaction with LSD1/co-REST. A previous study revealed that upregulation of HOTAIR and miR-196a was associated with high-risk GISTs, although the exact mechanism was not reported [4]. HOTAIR has been known to play an important role in various cancers, and genome-wide analysis indicates that it may, at least in part, be mediated by HOTAIR targeting the PRC2 complex to specific genes. Although many studies showing the new mechanism of HOTAIR in carcinogenesis have been performed since the previous paper, it has not been further studied in GISTs. Herein, we investigated the mechanistic role of HOTAIR in the onset and progression of gastric GISTs.

## RESULTS

### HOTAIR is upregulated in a high-risk GIST

**Figure 1:**
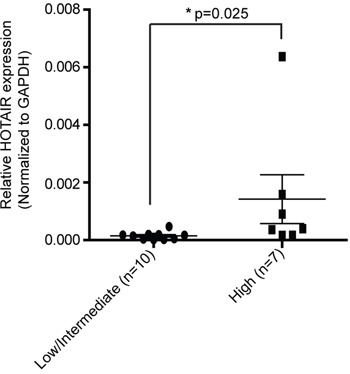
HOTAIR expression is higher in high-risk GISTs than in low/intermediate-risk GISTs The expression level of HOTAIR was determined by qRT-PCR in 17 surgically resected tissues using the 2-ΔΔct method. Data shown represent the mean ± SEM. The asterisk denotes a statistically significant difference between high-risk and low/intermediate-risk GISTs (*P ≤ 0.05).

It has already been shown, that the expression of HOTAIR is upregulated in gastric cancer tissues compared to adjacent normal tissues [5]. To further investigate the impact of HOTAIR on GISTs, the latter were divided into two groups based on the degree of malignancy. We had data for 17 cases of GISTs including nine low-risk GISTs, one intermediate-risk GIST, and seven high-risk GISTs. To investigate the association of HOTAIR with the malignant potentials of GIST, we analyzed the expressions of HOTAIR in low/intermediate-risk and high-risk GISTs. We observed that the expression of HOTAIR was significantly higher in the high-risk GISTs than in low/intermediate-risk GISTs (p = 0.025; Figure 1A). Taken together, our results suggest that HOTAIR expression is related to the malignancy of GISTs.

### Dysregulation of HOTAIR suppresses cell proliferation in GIST cells

**Figure 2:**
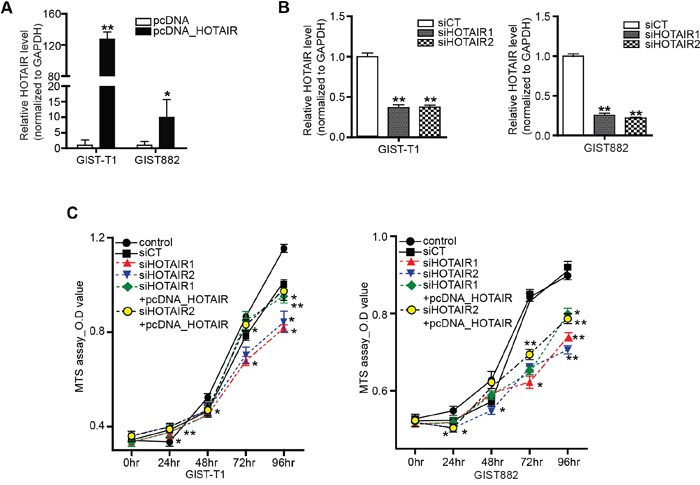
Inhibition of HOTAIR suppresses GIST cell proliferation HOTAIR expression was detected by qRT-PCR in GIST-T1 and GIST882 cells transfected with **A.** pcDNA-HOTAIR and **B.** siHOTAIRs. The bars represent relative HOTAIR expressions. **C.** Cell viability was analyzed via a MTS assay. siHOTAIR1 and siRHOTAIR2 were compared to scrambled controls. siHOTAIR1+pcDNA-HOTAIR and siHOTAIR2+pcDNA-HOTAIR were compared to siHOTAIR1 and siRHOTAIR2, respectively. Data are expressed as mean ± SEM (n=3). The asterisk denotes a statistically significant difference compared to each companions (*P ≤ 0.05; ** *P* ≤ 0.01).

Having confirmed the upregulation of HOTAIR in high-risk GISTs, we then focused on its functional mechanism. To this end, we used two different GIST cell lines: GIST-T1 (a heterozygous mutation in KIT exon 11), and GIST882 (homozygous mutation in KIT exon 13). In both cell lines, HOTAIR was either overexpressed (10 to 150 times) using vector pcDNA-HOTAIR (Figure 2A), or downregulated (50 % to 60% of that in the siCT negative control) using two different siRNAs targeting HOTAIR (siHOTAIRs; Figure 2B). An MTS assay revealed that cell proliferation decreased significantly after siHOTAIRs treatment at different time points compared to control and siCT-treated cells. In particular, growth gaps of 72 and 48 hours were observed between siHOTAIRs- and siCT-treated cells with statistical significance, respectively (Figure 2C). The cell proliferation, in pcDNA-HOTAIR treatment after siHOTAIRs, was restored in comparison to siHOTAIRs treatment with statistical significance (Figure 2C). These results show that cell proliferation was inhibited by siHOTAIRs and restored by pcDNA-HOTAIR, suggesting that HOTAIR might be associated with cell death.

### HOTAIR regulates methylation of PCDH10

**Figure 3:**
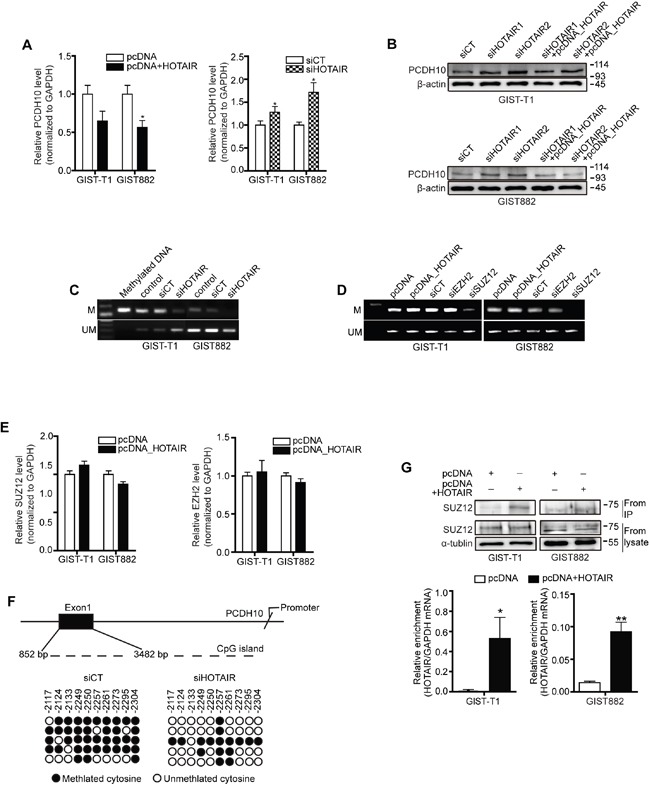
HOTAIR controls the methylation of PCDH10 PCDH10 expression was measured in GIST-T1 and GIST882 cell lines after treatment with pcDNA-HOTAIR (**A.**; left panel), and in GIST882 cells after treatment with siHOTAIRs (A, right panel). The data shown are representative of three independent experiments. The bars represent relative PCDH10 mRNA levels. **B.** PCDH10 protein level was determined via Western blot in GIST-T1 and GIST 882 cells. **C.** The methylation of PCDH10 after treatment with siHOTAIRs was measured via MS-PCR. **D.** The methylation of PCDH10 was measured after treatment with siEZH2 or siSUZ12. M, methylated DNA; UM, unmethylated DNA. **E.** mRNA of SUZ12 and EZH2 was measured via qRT-PCR after transfection of pcDNA-HOTAIR. NS: not significant. **F.** DNA from GIST-T1 treated with siCT or siHOTAIRs was treated with bisulfite, and the promoter regions in axon 1 were amplified by PCR and cloned. Each circle represents a CpG and filled ovals indicate methylation. Open ovals indicate un-methylation. **G.** The interaction between SUZ12 and HOTAIR was confirmed via RIP analysis. The bars present relative enrichment. Data are expressed as mean ± SEM. The asterisk denotes a statistically significant difference compared to pcDNA or scrambled control (*P ≤ 0.05, **P ≤ 0.01).

Previous genome-wide and microarray studies reported changes in the expression of specific target genes following HOTAIR overexpression [4, 6]. Thus, we tested the expression of several candidate genes as mentioned [4] and carried out a Nano String nCounter Gene Expression Assay to additional pathway analysis (Supplementary table S1 and S2). In particular, PCDH10 expression was approximately 40% to 50% downregulated in GIST-T1 and GIST882 cells transfected with pcDNA-HOTAIR compared to vector alone (Figure 3A, left) in our study. In contrast, the treatment of siHOTAIRs increased the transcriptional level of PCDH10 (Figure 3A, right). Furthermore, PCDH10 protein was also upregulated by siHOTAIRs and downregulated by pcDNA-HOTAIR in both GIST cell lines (Figure 3B). It has been reported that in many cancers, including gastric cancer, methylation determined the epigenetic silencing of PCDH10 [7–12]. We analyzed PCDH10 methylation by performing a methylation-specific PCR after silencing or overexpressing HOTAIR in GIST cells. Methylated DNA was used as a positive control. Methylation of PCDH10 was significantly decreased by HOTAIR silencing in both GIST-T1 and GIST882 cells (Figure 3C). In contrast, un-methylation level was increased or remained unchanged (Figure 3C). Next, we analyzed PCDH10 methylation by selectively silencing two PRC2 subunits. Methylation of PCDH10 was decreased by SUZ12, though EZH2 silencing was not affected (Figure 3D). The overexpression of HOTAIR by pcDNA-HOTAIR affected neither SUZ12 nor the EZH2 mRNA level (Figure 3E). Furthermore, the MS-PCR result was confirmed by bisulfite genomic sequencing (BGS), which indicated that methylated alleles are located at exon 1 on PCDH10 promoter CpG islands. The status of methylation was lower in siHTOAIRs than in siCT in BGS (Figure 3F). These findings support those of Gupta et al., who reported that HOTAIR overexpression did not alter the levels of PRC2 subunits, and instead led to high occupancy of PCDH10 by SUZ12 yet not EZH2 [6]. To clarify the interaction of HOTAIR with SUZ12, we performed RNA immunoprecipitation (RIP). As shown in the results, SUZ12 expression was detected and increased in pcDNA-HOTAIR compared to pcDNA in both cell lines (Figure 3G). HOTAIR enrichment was also increased in pcDNA-HOTAIR compared to pcDNA (Figure 3G). To this end, we report that HOTAIR could play a key role in PCDH10 regulation through SUZ12-specific methylation and could thus epigenetically regulate GISTs.

### Restoration of PCDH10 by 5-Aza treatment stimulated HOTAIR silencing induced-suppression of cell invasiveness and migration

**Figure 4:**
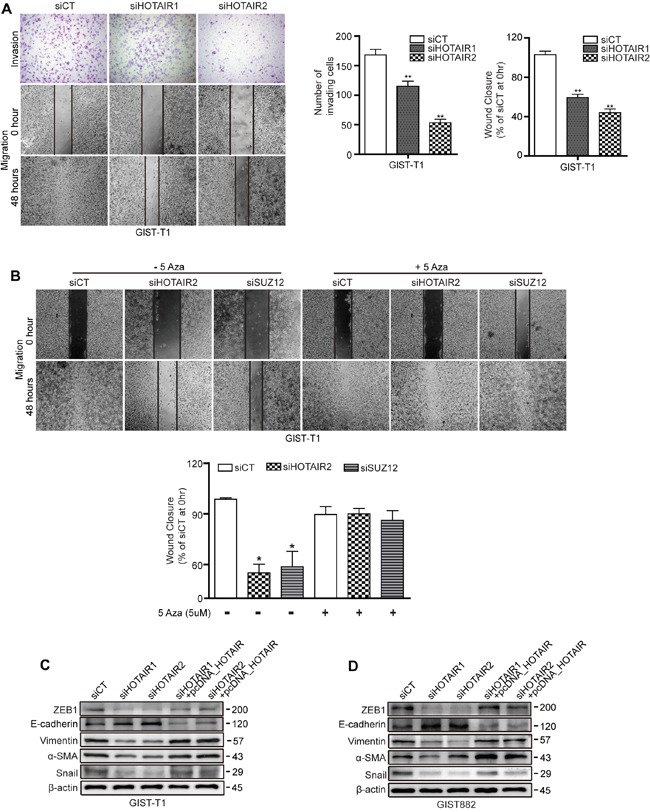
siHOTAIRs suppress cell invasiveness and migration capacity **A.** Matrigel-transwell invasion (upper row) and wound healing assays (second and third rows) were performed using GIST-T1 cells transfected with siHOTAIRs, and were observed after 48 hours. The bar graph indicates the number of invading cells and percentage of wound closures. **B.** 5-Azacytidine was treated after transfection with siCT, siHOTAIR or siSUZ12. **C** and **D.** GIST cells transfected with siCT, siHOTAIRs, or siHOTAIR+pcDNA-HOTAIR were analyzed for EMT markers via Western blot. Figures are representative of three independent experiments. Data are expressed as mean ± SEM (n=3). The asterisk denotes a statistically significant difference compared to scrambled controls (*P ≤ 0.05, **P ≤ 0.01).

In our previous study, we reported that HOTAIR silencing suppressed invasion/migration in gastric cancer cells. Here, we investigated whether HOTAIR was associated with invasiveness and migration in GIST cells. As shown for GIST-T1 cells, invasiveness and wound closure were suppressed by siHOTAIRs compared to siCT controls (Figure 4A). Next, we investigated whether methylation of PCDH10 controls migration of GIST cell by using methyl-transferase inhibitor. 5-Azacytidine (5-Aza) as a methyl-transferase inhibitor was administered after siHOTAIRs or siSUZ12. After 48 hours, 5-Aza diminished the delayed wound closure induced by siHOTAIRs or siSUZ12 and restored migration to levels smilar to siCT (Figure 4B). As GISTs are the most common mesenchymal tumor, we looked at the effect of HOTAIR silencing on the levels of epithelial-mesenchymal transition (EMT)-related markers in cell lines originating from GISTs. In both GIST-T1 and GIST882, the protein level of vimentin, a major cytoskeletal component of mesenchymal cells, was noticeably lower in siHOTAIRs-treated cells than in siCT-treated cells (Figure 4C and 4D). In addition, levels of other markers including ZEB1, smooth muscle actin and Snail decreased on siHOTAIR treatment compared to siCT controls. However, the low protein levels were restored by overexpressing HOTAIR using pcDNA-HOTAIR. In contrast, protein levels of E-cadherin, an epithelial marker, increased following siHOTAIRs treatment, and could be restored by pcDNA-HOTAIR (Figure 4C and 4D).

### HOTAIR silencing induces apoptosis and cell cycle arrest

**Figure 5:**
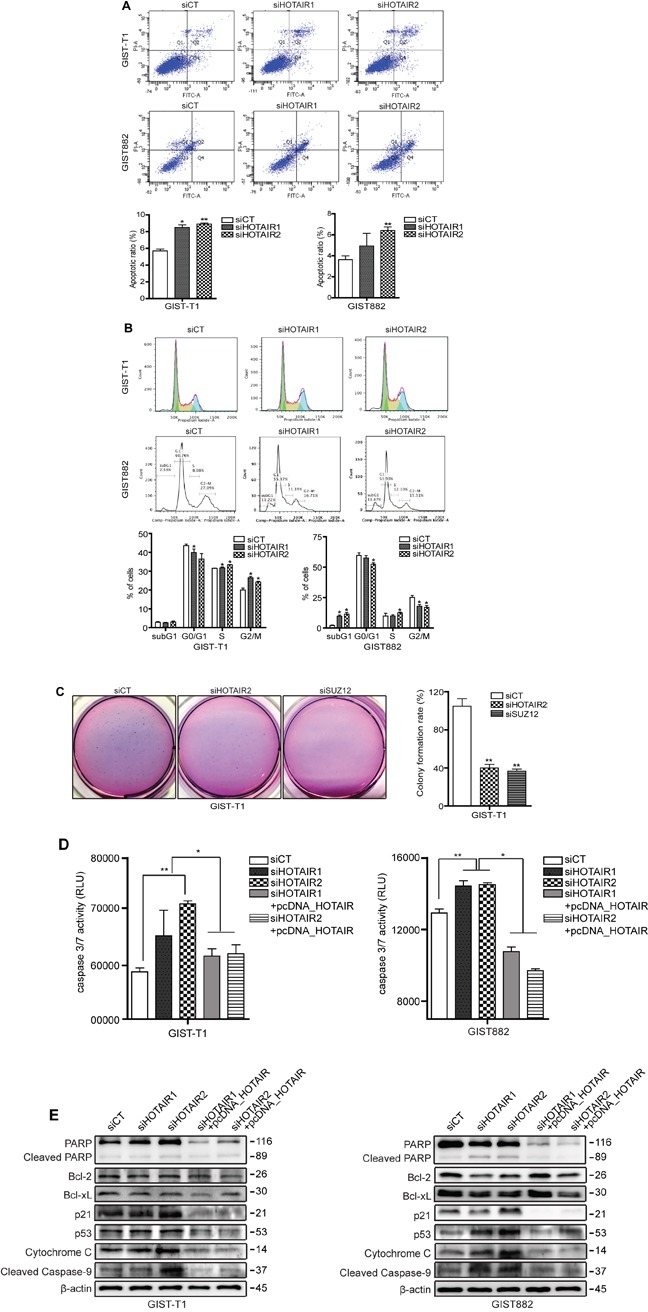
siHOTAIRs induce apoptosis and cell cycle arrest GIST-T1 and GIST882 cells were transfected with siCT or siHOTAIRs. Apoptosis and cell cycle analysis were performed using PI/Annexin-V **A.** and PI **B.** staining. The bar represents ratio of apoptotic phase (A) and percentage of cells (B). siHOTAIR1 and siHOTAIR2 were compared to scrambled control (siCT). **C.** Soft agar colony formation was performed after treatment with siHOTAIRs or siSUZ12. The bar represents the rate of colony formation. siHOTAIR2 and siSUZ12 were compared to scrambled control (siCT). **D.** A caspase -3 and -7 assay was measured via ELISA after treatment with siCT, siHOAIRs or pcDNA-HOTAIR. siHOTAIR1 and siRHOTAIR2 were compared to scrambled controls (siCT). siHOTAIR1+pcDNA-HOTAIR and siHOTAIR2+pcDNA-HOTAIR were compared to siHOTAIR1 and siRHOTAIR2, respectively. **E.** Apoptotic and cell cycle markers were detected in transfected GIST cells via Western blot. Data shown are representative of three independent experiments (*P ≤ 0.05; **P ≤ 0.005).

To follow up on our cell proliferation results, we examined the effect of HOTAIR on apoptosis and cell cycle progression. Staining with PI/Annexin V revealed a significant increase in the early-to-late apoptotic ratio in HOTAIR knockdown GIST cells (Figure 5A). Moreover, the high apoptotic ratio closely correlated with a cell cycle regulatory checkpoint mediated by HOTAIR silencing. Treatment with siHOTAIRs increased cell cycle arrest in G2/M phase, concomitantly with a reduction in the number of GIST-T1 cells in G0/G1 (Figure 5B). Meanwhile, the population of late apoptotic subG1 was significantly increased on siHOTAIRs treatment in GIST882 cells (Figure 5B), however this was not observed in GIST-T1cells.

Next, we performed a soft agar colony formation assay to confirm cellular anchorage-independent growth *in vitro*. The forming capability of colonies was significantly decreased in both siHOTAIR2 and siSUZ12 treatment compared to siCT (Figure 5C). Caspase in apoptosis-related activity plays a critical role in cell regulatory networks controlling cell death. We measured the activity of caspase-3 and -7 as executioner caspases. The activity of caspase3/7 in both GIST-T1 and GIST882 cells was increased by siHOTAIRs treatment while pcDNA-HOTAIR decreased the activity (Figure 5D). The same apoptotic profile was detected at a translational level in both GIST-T1 and GIST882 cells (Figure 5E). Accordingly, p21 and p53 protein levels were higher in siHOTAIR-transfected GIST-T1 cells compared to controls, leading to the downstream activation of cytochrome c and cleaved caspase-9 (Figure 5E). Finally, cleavage of poly (ADP-ribose) polymerase (PARP) was also induced by siHOTAIRs. In GIST882, protein levels of two anti-apoptotic markers, Bcl-2 and Bcl-xL, were decreased by siHOTAIRs, while those of p53, cytochrome c and cleaved PARP were increased compared to the control cells (Figure 5E). This pattern was restored upon HOTAIR overexpression using pcDNA-HOTAIR in both GIST-T1 and GIST882 cells (Figure 5E). A microarray confirmed that p53 was downregulated by 9.0-fold in HOTAIR-overexpressing GIST-T1 cells.

## DISCUSSION

**Figure 6:**
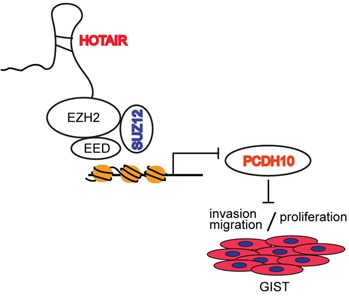
Promoter methylation of PCDH10 by HOTAIR regulates proliferation and invasion/migration LncRNA HOTAIR regulates invasion/migration and proliferation by suppressing promoter methylation of PCDH10.

Functional and clinical studies in a variety of cancers have correlated the loss of lncRNA HOTAIR regulation with carcinogenesis and metastasis [6, 13–16]. In this study, we report that HOTAIR expression was elevated in high-risk malignancy samples from frozen GIST tissues. This finding confirms previous speculations, as it implies that HOTAIR contributes to carcinogenesis and malignancy, including invasion and migration, in GISTs. In this study, we provide mechanistic evidence that epigenetic disruption of PCDH10 by HOTAIR induces carcinogenesis and invasiveness, and upregulates the transcriptional factor p53, which is a direct target of PCDH10. HOTAIR has been shown to regulate the expression of target genes by binding to the PRC2 complex, whose methyltransferases mediate the trimethylation of H3K27 [6]. We suggest that the epigenetic disruption of PCHD10, a PRC2-targeted gene, by HOTAIR blocks apoptosis and promotes metastasis in GISTs. Crucially, we show that HOTAIR controls the apoptosis of GIST cells and that this regulatory step is mediated by the methylation of PCDH10 by HOTAIR (Figure 6).

We found PCDH10, a tumor suppressor gene, to be downregulated in high-risk GIST tissue and cells overexpressing HOTAIR. PCDH10 is a member of the protocadherin gene family, and is silenced in, among others, colorectal, gastric, and non-small lung cancers [7–9, 11, 12]. The frequent silencing of PCDH10 in cancer is ascribed to the aberrant hypermethylation of its promoter and, consequently, altered signal transduction, resulting in the failure to suppress invasion and tumor progression. Nevertheless, PCDH10 had never before been studied in relation to GISTs. Here, the PCDH10 protein level decreased significantly in both GIST-T1 and GIST882 cells after HOTAIR overexpression and consequent HOTAIR-induced DNA methylation of PCDH10. This finding was confirmed by HOTAIR silencing and HOTAIR overexpression experiments, respectively. Furthermore, the methylation, and transcription of PCDH10 were restored by pcDNA-HOTAIR in GIST cells previously treated with siRHOTAIRs. Our findings were supported by recent papers caliming that HOTAIR can modulate the methylation of target genes [17–20].

In this study, the methylation of PCDH10 by HOTAIR was specifically dependent on SUZ12. We found that HOTAIR bind SUZ12 by RNA immunoprecipitation. siHOTAIRs and siSUZ12 each downregulated the methylation of PCDH10. These findings were confirmed by MS-PCR and BGS. More importantly, 5-Aza diminished the phenomenon exhibited by the knockdown of HOTAIR and SUZ12. This finding is supported by a previous report in which HOTAIR heterochromatin occupancy on PCDH10 showed high affinity in the presence of SUZ12, though not in the presence of EZH2 [6]. In light of these results, the methylation of PCDH10 and its control by the HOTAIR/SUZ12 pathway represents novel molecular mechanisms of malignancy, which play important roles in GIST pathogenesis. In our study, the knockdown of HOTAIR and SUZ12 did not completely block the methylation of PCDH 10. GIST882 and GIST-T1 cells showed only slight differences in dependency on the HOTAIR/SUZ12 pathway and its related biologic phenomena. There might also be an alternative pathway to control PCDH10 methylation-independent HOTAIR/SUZ12 in GISTs.

In this study, the silencing of HOTAIR and SUZ12 decreased cell proliferation and colony formation, respectively. The silencing of HOTAIR promoted apoptosis and, induced G2/M arrest in GIST-T1 cells, and it increased the population of subG1 in GIST882 cells. The restoration of apoptosis by pcDNA-HOTAIR in these samples confirmed our findings. Although the apoptotic ratio increased in both GIST-T1 and GIST882 cells, the cell cycle analysis showed clear cell type-specific differences as dependency on HOTAIR/SUZ12. This finding may be explained by the specificity of the KIT mutation in the GIST cell type [21]. Levels of apoptotic protein markers were high in both GIST-T1 and GIST882 cells, particularly for cell cycle regulatory checkpoint markers p21 and p53 in GIST-T1.

In our study, RNAi-mediated HOTAIR and SUZ12 silencing suppressed invasion and migration in GIST-T1 cells and led to decreased expression of mesenchymal markers, such as Snail. siHOTAIRs-induced migration was diminished by treatment with 5-Aza and pcDNA-HOTAIR. This finding confirm that migration and invasion activity is controlled by HOTAIR and its related methylation of target genes. In our experimental findings HOTAIR showed involvement in both apoptosis and cell migration and invasion. However, attention should also be given to the analysis of migration and invasion as apoptosis itself can affect cell migration. Furthermore, Kalluri R. et al, recently reported that Snail- or Twist-induced EMT was not rate-limiting for invasion or migration in their pancreatic cancer mice model; instead, EMT was found to result in the suppression of cancer cell proliferation [22] This paper presented new concept of EMT in terms of invasion and proliferation.

Finally, we identified a novel regulatory mechanism involving the methylation of PCHD10 by HOTAIR/SUZ12 in GISTs and demonstrated that the PCDH10/HOTAIR pathway modulated cell proliferation and the invasion and migration of GIST cells. These results suggest that therapeutics controlling the PCDH10/HOTAIR pathway may attenuate GIST progression and metastasis. PCDH10 expression, the degree of PCDH10 methylation, and the level of HOTAIR represent potential markers of malignancy in GIST.

Although a larger number of GIST samples will be required to further validate our findings, the present data suggest that the methylation of PCHD10 by HOTAIR may yield new insights in the pathogenesis and malignant potentials of GISTs, offering more aggressive surveillance and treatment.

## MATERIALS AND METHODS

### Patients and tissue samples

Fresh gastrointestinal stromal tumor samples were obtained from 17 patients who underwent surgical resection for gastric cancer at Severance Hospital, Yonsei University College of Medicine, Seoul, Korea. All samples were frozen in liquid nitrogen immediately after resection and stored at −80°C until further use. The specimens were obtained from the archives of the Department of Pathology at Yonsei University and from the Liver Cancer Specimen Bank of the National Research Resource Bank Program (Korea Science and Engineering Foundation, Ministry of Science and Technology). The use of these tissues for research purposes was authorized by the Institutional Review Board of Yonsei University College of Medicine (4-2015-0178). The median size of all GISTs was 6.65 cm (3-17 cm) and the mitotic count ranged from 1 to 49 with a median 5. The median sizes of high-risk GISTs and low/intermediate-risk GISTs were 7.15 cm and 6.25 cm, respectively. The median numbers of mitosis of high-risk GISTs and low/intermediate-risk GISTs were 21.5 and 3, respectively.

### Cell lines and cell culture

GIST-T1 cells were commercially purchased (Cosmo Bio, Tokyo, Japan) and GIST882 cells were kindly provided by Dr. Hogeun Kim. The cells were cultured in DMEM medium (Thermo Scientific, Rockford, IL, USA) supplemented with 10% fetal bovine serum (FBS), 1% penicillin and streptomycin. Cells were maintained in a humidified atmosphere of 5% CO_2_ in air at 37°C.

### Small interfering RNA (siRNA) transfection

GIST-T1 and GIST882 cells were plated on 6-well culture plates and incubated at 37°C overnight. After 24hours, the cells were transfected with siRNAs targeting HOTAIR and a Stealth RNAisiRNA negative control (siCT; Invitrogen, Carlsbad, CA, USA) using Lipofectamine 2000 (Invitrogen) following the manufacturer's protocol. The following HOTAIR siRNAs target sequences were used: siHOTAIR1, sense: 5′-GAACGGGAGUACAGAGAGAUU-3′, antisense: 5′-AA UCUCUCUGUACUCCCGUUC-3′; siHOTAIR2, sense: 5′-CCACAUGAACGCCCAGAGAUU-3′, antisense: 5′-AA UCUCUGGGCGUUCAUGUGG-3′ [6].

### Total RNA extraction, reverse transcription and quantitative real-time PCR (qRT-PCR)

Total RNA extraction from GISTs tissues and cell lines was performed using TRIzol reagent (Invitrogen). For cDNA synthesis, 2.0 μg of total RNA was reverse transcribed using Superscript^TM^II (Invitrogen) following the manufacturer's protocol. The relative expression of HOTAIR was measured via qRT-PCR using iQ SYBR Green Supermix (Applied Biosystems Inc., Carlsbad, CA, USA), and analyzed on a Roche LightCycler480 Real-Time PCR System (Roche, Penzberg, Germany). The qRT-PCR ct value was calculated with the 2-ΔΔct method and normalized to GAPDH. The qRT-PCR target sequences for HOTAIR, PCDH10, SUZ12 and EZH2 were used as described [6, 23, 24].

### Methylation-specific PCR (MS-PCR) and bisulfite genomic sequencing (BGS)

Genomic DNA was extracted from GIST-T1 and GIST882 cells using the DNeasyBlood&Tissue kit (Qiagen, Valencia, CA, USA). DNA bisulfate conversion was performed with the EZ DNA Methylation-Gold Kit™ (ZYMO Research, Irvine, CA, USA) followed by a MS-PCR. The following MS-PCR primers for PCDH10 were used as described: Methylation (forward: GTTAGGGAGGATGGATGTAAGTATC, reverse: GCG AAATAAAAACAATAAAACGAC) and; un-methylation (forward: GTTAGGGAG GATGGATGTAAGTATT, reverse: CCCACA AAATAA AAACAATAA AA AA) [9]. The amplified DNA was cloned into TOPO cloning vector (Invitrogen) and sequenced using DNAMAN software.

### Cell proliferation assay and caspase assay

GIST-T1 and GIST882 cells were transfected with 50nM siHOTAIRs or pcDNA-HOTAIR from 0 to 96 hours. Cell proliferation was determined by the CellTiter 96® AQueous One Solution Cell Proliferation Assay (MTS assay; Promega, Madison, WI, USA) in 96-well culture plates at different time points. After transfection with siHOTAIRs or pcDNA-HOTAIR cells were allowed to react with the MTS reagent for 1 hour in the dark. The ensuing formazan product was measured via enzyme-linked immunosorbent assay (ELISA). For caspase analysis, caspase -3 and -7 reagent was added after treatment with siHOTAIRs or pcDNA-HOTAIR in the multiwell plates of a 96-luminometer. Luminescence activity was measured using a Luminescence Microplate Reader (Molecular Devices Co., Sunnyvale, CA, USA).

### HOTAIR-overexpressing plasmid construction

To overexpress HOTAIR in GISTs, we used human HOTAIR cDNA (Addgene, Plasmid #26110, Cambridge, MA) as reported previously [25]. The HOTAIR cDNA was amplified by PCR and the resulting product was inserted in the pcDNA3.1 vector (Addgene, Plasmid #47388) using TOPO cloning (Invitrogen) following the manufacturer's protocol. The HOTAIR expressing vector was sequenced and analyzed using Macrogen (Macrogeninc., Seoul, Korea). GIST cells were transfected with 1ug pcDNA3.1-HOTAIR (pcDNA-HOTAIR) using Lipofectamine 2000 (Invitrogen) for 24 hours.

### RNA immunoprecipitation (RIP)

GIST cells were transfected with pcDNA or pcDNA-HOTAIR. For immunoprecipitation of endogenous RNP complex, the cells were lysed with nuclear isolation buffer (1.28 M sucrose, 40 mM Tris-HCl [pH7.5], 20 mM MgCl2, 4% Triton X-100) and resuspended in RIP buffer (150 mM KCl, 25 mM Tris-HCl [pH7.4], 5 mM EDTA, 0.5 mM DTT, 0.5% NP40, 1U/ul RNAase inhibitor, protease inhibitor). The shearing of chromatin was mechanically conducted using a Dounce homogenizer with 15-20 strokes. After centrifugation, SUZ12 antibody (2ug) was added to supernatant (600-800ug) and incubated for 2 h at 4°C with gentle rotation. After incubation, Magna Chip protein A magnetic beads (Merck Millipore, Darmstadt, Germany) was added and incubated for 2 hrs at 4°C with gentle rotation. The pellet was washed with RIP buffer and repeated for a total of three RIP washes. After washing, each sample was lysed with TRizol reagent or eluted in SDS, and then analyzed on Western blot.

### Apoptosis and cell cycle analysis

Transfected GIST-T1 and GIST882 cells were washed with phosphate buffered saline (PBS, Thermo Scientific) and resuspended in 1X binding buffer (BD Biosciences, San Jose, CA, USA). Fluorescein isothiocyanate (FITC) Annexin V and propidium iodide (PI) staining were performed using the FITC Annexin V detection kit (BD Biosciences) following the manufacturer's protocol. For cell cycle analysis, GIST-T1 and GIST882 cells were transfected with 50nM siHOTAIRs or siCT, washed with PBS (Thermo Scientific) and fixed with 75% ethanol at −20°C overnight. The nuclei of fixed cells were stained with 50 mg/ml PI (Sigma, Saint Louis, MO, USA) in the dark. Cell cycle phases were analyzed via flow cytometry (BD Biosciences).

### Invasion and migration assay

For the invasion assay, GIST-T1 cells were transfected with siRNAs targeting HOTAIR and replated on BD BioCoat trans-wells (BD Biosciences) according to the manufacturer's protocol. The invading cells on the membrane were examined and counted under a bright field microscope. An average value was calculated by counting the invading cells within five random portions of the membrane. For the migration assay, GIST-T1 cells were transfected with si-HOTAIR1, si-HOTAIR2, si-SUZ12, or control si-CT. When the cells formed an approximately 90% confluent monolayer, scratch wound healing was induced using a P-20 micropipette tip. For analysis methylation inhibition, 5-Azacytidine was treated with 5uM. The widths of the scratched cells were measured at 0 and 48 hours under bright field microscopy.

### Soft agar colony formation assay

To analyze tumorigenicity in *vitro*, base and top agarose were coated in 6-well cultured plates. 1.5ml of 2X DMEM containing 1% UltraPure Low Melting Point Agarose (Gibco, Rockville, MD, USA) was added into each well as a base. After solidification, the transfected cells were resuspended in 2X DMEM containing 0.7% UltraPure Low Melting Point Agarose as a top. The plates were maintained in a humidified atmosphere of 5% CO_2_ in air at 37°C for 2-3 weeks. Colonies were stained and measured under bright field microscopy.

### Western blot

Transfected cells were lysed in 1X RIPA buffer (Cell Signaling Technology, Danvers, MA, USA) containing protease inhibitors. The following primary antibodies were used for Western blot analysis: epithelial marker E-cadherin (1:1000, BD Biosciences, 610181), mesenchymal marker N-cadherin (1:1000, BD Biosciences, 610920), ZEB1 (1:1000, Cell Signaling Technology, #3396), vimentin (1:200, Santa Cruz Biotechnology, Dallas, TX, USA, sc-373717), smooth muscle actin (1:200, Santa Cruz Biotechnology, sc-53142), Snail (1:1000, Cell Signaling Technology, #3895), cytochrome c (1:1000, Cell Signaling Technology, #12959), PARP (1:1000, Cell Signaling Technology, #9542), bcl-2 (1:1000, Cell Signaling Technology, #2870), bcl-xl (1:1000, Cell Signaling Technology, #2764), p21 (1:200, Santa Cruz Biotechnology, sc-397), p53 (1:200, Santa Cruz Biotechnology, sc-126), cleaved caspase-9 (1:1000, SUZ12 (1:1000, Abcam, Cambridge, MA, ab12073) PCDH10 (1:1000, Thermo Scientific, Cell Signaling Technology, #7237), PA5-31042), alpha-Tublin (1:1000, AbFrontier, Seoul, Korea, LF-PA0146A) and β-actin (1:5000, Bioworld Technology, Louis Park, MN, USA, AP0060).

### Statistical analysis

All data were analyzed for continuous and categorical variables and are presented as the mean ± standard error. Statistical tests included the *t*-test, χ2 test, and Fisher's exact test. The expression of HOTAIR in GISTs was categorized as high-risk or low/intermediate-risk based on median HOTAIR expression. All statistical procedures were performed in SPSS for Windows (version 18.0; SPSS Inc., Chicago, IL, USA).

## SUPPLEMENTARY TABLES




