# Supplementary material for: Promoter methylation of PCDH10 by HOTAIR regulates the progression of gastrointestinal stromal tumors
Source: Oncotarget. 2016 Sep 21;7(46):75307–18. doi: 10.18632/oncotarget.12171 (PMC5342742; doi:10.18632/oncotarget.12171)
Supplement: Supplementary file 1 [file oncotarget-07-75307-s001.pdf]

Promoter methylation of PCDH10 by HOTAIR regulates the progression of gastrointestinal stromal tumors

SUPPLEMENTARY TABLES

Supplementary Table S1: HOTAIR-induced PRC2 target gene expression in HOTAIR silenced-GIST cells

GeneSymbol	GeneName	Fold change (compared to siCT)
RPS6KA2	ribosomal protein S6 kinase, 90kDa, polypeptide 2	1.041
JAM2	junctional adhesion molecule 2	1.154
PCDH10	protocadherin 10	1.925

Supplementary Table S2: Cancer-related target gene expression in HOTAIR overexpressing GIST-T1 cell

See Supplementary File 1
